# Regulatory effects and potential therapeutic implications of alarin in depression, and arguments on its receptor

**DOI:** 10.3389/fpsyt.2022.1051235

**Published:** 2022-11-25

**Authors:** Endeshaw Chekol Abebe, Misganaw Asmamaw Mengstie, Mohammed Abdu Seid, Tadesse Asmamaw Dejenie

**Affiliations:** ^1^Department of Medical Biochemistry, College of Health Sciences, Debre Tabor University, Debre Tabor, Ethiopia; ^2^Department of Physiology, College of Health Sciences, Debre Tabor University, Debre Tabor, Ethiopia; ^3^Department of Medical Biochemistry, College of Medicine and Health Sciences, University of Gondar, Gondar, Ethiopia

**Keywords:** depression, antidepressant effect, alarin receptor, therapeutic implication, alarin

## Abstract

Alarin is a pleiotropic peptide involved in a multitude of putative biological activities, notably, it has a regulatory effect on depression-like behaviors. Although further elucidating research is needed, animal-based cumulative evidence has shown the antidepressant-like effects of alarin. In light of its regulatory role in depression, alarin could be used as a promising antidepressant in future treatment for depression. Nevertheless, the available information is still insufficient and the therapeutic relevance of alarin in depression is still of concern. Moreover, a plethora of studies have reported that the actions of alarin, including antidepressant activities, are mediated by a separate yet unidentified receptor, highlighting the need for more extensive research. This review focuses on the current understanding of the regulatory effects and future therapeutic relevance of alarin on depression, and the arguments on its receptors.

## Introduction

Depression is a common mental illness that presents with an abnormal lowering of mood (feeling sad, irritable, and empty), a loss of pleasure or interest in activities, feelings of excessive guilt or low self-esteem, and feelings of hopelessness about the future, anxiety, sleep disturbances, changes in appetite or body weight, feeling tired, thoughts of suicide, sexual dysfunction, and cognitive impairments (1). It is one of the most widespread health threats to humans, affecting about 4.4% of the global population (2). It is also a leading cause of disability worldwide, contributing significantly to health, social, and economic problems (3, 4). Although the pathogenesis of depression is not well elucidated, the interplay between genetic and psychosocial predisposing factors has been suggested to be involved (5, 6). Numerous medications for the treatment of depression are currently available, but most of them are associated with low therapeutic response rates as well as adverse effects, prompting researchers to look for a novel treatment option (7). In recent years, several neuropeptides, such as the galanin neuropeptide family, have been discovered to have promising therapeutic relevance in depressive mood disorders (8, 9). Galanin, the ancestral peptide of the galanin family, has been shown to be implicated in depression and to have therapeutic value (10). Alarin, another member of the galanin family, has also recently been demonstrated to play a pivotal regulatory role in depression-like behaviors (11).

Alarin is a 25-amino acid containing biologically active peptide from the family of galanin, which was discovered for the first time 16 years ago in the gangliocytes of human neuroblastic tumors (12). It is a splice variant of galanin-like peptide (GALP) that results from alternative splicing of the GALP gene through exon three exclusion (13). Its name was termed after its N-terminal alanine and C-terminal serine (12). In healthy individuals, the normal plasma concentration of alarin is 0.37 ±0.10 μg/L (14). Alarin is broadly distributed in the central nervous system (CNS) and different peripheral tissues of various species like skin, eyes, the gastrointestinal tract, and the endocrine glands.

According to numerous rodent studies, the alarin peptide is distributed in a wide range of nuclei in the brain of mice and rats, including the mitral cell layer of the olfactory bulb, the accessory olfactory bulb, the medial preoptic area, the amygdala, and the bed nucleus of the stria terminalis (15–18). Besides, alarin is markedly expressed in a number of nuclei in the hypothalamus, such as paraventricular nucleus (PVN), dorsomedial nucleus (DMN), and the arcuate nucleus (ARC), ventromedial nucleus (VMN), and lateral hypothalamus (15, 16, 19). Moreover, this neuropeptide is expressed in the locus coeruleus (LC), and locus subcoeruleus of the midbrain and hindbrains, as well as the trigeminal complex, the ventral cochlear nucleus, the facial nucleus, and the epithelial layer of the plexus choroides in the brain (15, 19). Based on a clinical study by Eberhard et al. alarin is present in a variety of nuclei in human brain as demonstrated from medium to high-intensity alarin-like immunoreactivity (LI) in all choroid plexus tumors, in the majority of ependymomas, and the minority of astrocytomas, meningiomas, and tumors of the cranial nerves (18). However, clinical studies demonstrating the tissue distribution of alarin in human are limited, which warrants further research to identify the difference in the expression of alarin and its receptor among various species.

Alarin is a pleiotropic peptide with multifaceted biological functions (15, 16, 19, 20). It is involved in a series of important physiological effects, like the regulation of energy homeostasis, feeding behavior, body weight, and body temperature. Central injection of alarin into PVN has been shown to increase acute food intake and body weight in animals (15, 16, 19, 20). Alarin also has thermoregulatory effects that elicit significant fever-like hypermetabolic/hyperthermic thermoregulatory responses in rats (21). Additionally, this peptide plays a critical function in glucose metabolism by stimulating insulin-mediated glucose uptake in a variety of tissues in rodents (16, 20, 22). Moreover, alarin has a reproductive function that promotes the secretion of sex hormones such as luteinizing hormone (LH) (15, 20). In addition, this peptide exhibits antiedema, anti-inflammatory, and vasoconstrictive effects to maintain normal eye and skin health, antifibrinolytic activity, and antioxidant properties to prevent cardiac fibrosis, as well as antimicrobial activity against gram-negative bacteria (23–25). More importantly, it has been demonstrated that alarin exhibits antidepressant-like activity in various animal studies (11, 26–28). Although alarin has a variety of biological activities, the specific receptors that mediate these actions are still unknown. This review primarily points out the current understanding regarding the regulatory role of alarin in depression and its future potential therapeutic implications. Besides, the article provides a synopsis of the arguments concerning the alarin-specific receptor.

## Role of alarin in depression and its potential therapeutic implications

### Overview of the pathophysiology of depression

Depression is a seriously debilitating disorder that is thought to develop as a result of a constellation of genetic and psychosocial factors (5, 6). Numerous genomic studies indicate that depression is a heritable psychiatric disorder associated with a complex genetic architecture and several genetic variants (29). Although there is no solid evidence elucidating the pathophysiological mechanism underlying depression, a number of neurobiological hypotheses have been postulated in the past few decades, including the monoamine hypothesis, the neurotrophic hypothesis, the GABAergic hypothesis, the glutamatergic hypothesis, and the alteration of the hypothalamic-pituitary-adrenal (HPA) axis (30–34). The monoamine hypothesis, which entails the catecholamine hypothesis and the indolamine hypothesis, posits that the underlying pathophysiological basis of depression is a deficiency of the neurotransmitters serotonin, norepinephrine (NEP), or dopamine (DA) produced by the serotonergic, noradrenergic, and dopaminergic neurons in the monoaminergic systems, respectively (35). The catecholamine hypothesis is based on the findings that monoamine oxidase inhibitors (MAOIs) and tricyclic antidepressants (TCAs), which boost the levels and transmission of catecholamines, such as NEP and DA, exhibit antidepressant effects. This indicates that the depletion of catecholamines in the brain is linked to the pathomechanism of depression. Additionally, dysregulation of catecholamine transmission is postulated to be involved in depression (30, 35–38). The indolamine hypothesis, on the other hand, proposes a decrease in the activity of serotonin [or 5-hydroxytryptamine (5-HT)] in the brain as well as a deficiency in pre-synaptic and post-synaptic 5-HT receptors, particularly the 5-HT1A auto-receptor, which is a potential predisposing factor to depression (39–42).

According to the neurotrophic hypothesis, another pathophysiological hypothesis of depression, the downregulation of neurotrophins like brain-derived neurotrophic factor (BDNF) is related to persistent stress and depression. Numerous studies indicate that decreased hippocampal and cortical BDNF levels are significantly correlated with stress-induced depressive-like behaviors (43–45). Moreover, the GABAergic hypothesis suggests that the pathogenesis of depression is associated with reduced brain concentration of the inhibitory neurotransmitter γ-aminobutyric acid (GABA), as well as changes in its receptors (GABA-A receptors) mediating GABAergic inhibition in the prefrontal and occipital cortex (31, 32). GABAergic transmission, which is crucial for the regulation of hippocampal neurogenesis and neural maturation, plays a prominent role in the brain’s control of stress and represents a major vulnerability factor in depressive mood disorders. Persistent stress is thought to result in synaptic loss and changes in the interneuronal plasticity in circuits underlying affective and cognitive processes that contributes to the occurrence of depression. Recent reports have shown stress-induced alterations in the structure, connectivity, and neurochemistry of interneurons in depressed individuals. In particular, stress-induced plasticity of GABAergic inhibition occurs *via* changes in GABA synthesis, release, and the expression of specific GABA_*A*_-receptor subunits has been shown to be involved in the development of depression (46–49). A significant reduction in the concentration of GABA has been observed in the cortex, hypothalamus, and olfactory bulb as a result of chronic stress (50). Several lines of evidence indicate that chronic stress reduces GABAergic synaptic transmission in the ventral hippocampus due to the selective loss of hippocampal neurons, and leads to depression (33, 34).

On the other hand, the glutamatergic hypothesis argues that abnormalities in glutamatergic neurotransmission or glutamatergic toxicity are implicated in the biological mechanisms underlying depression. The glutamatergic hypothesis is based on the observation of quick and large antidepressant effects from blocking an ionotropic glutamate receptor called the glutamate N-methyl-D-aspartate (NMDA) receptor (51–53). Moreover, alteration of the HPA axis has been demonstrated to participate in the development of depression. According to this theory, the pathophysiology of depressive disorders is fundamentally linked to increased glucocorticoid secretion and an overactive HPA axis (54–57).

### Regulatory effects of alarin in depression

Accumulating evidence shows that some members of the galanin neuropeptide family are involved in mediating behavioral functions linked to stress and anxiety, suggesting their potential involvement in mood disorders like depression (58). Galanin, which is abundantly distributed in the hypothalamus and amygdala, is linked to stress response and depression as it is co-expressed with and modulates NEP and serotonin systems (59–63). Therapeutically, antagonists of galanin receptor (GalR) sub-type 1 (GalR1) and GalR3, as well as agonists of GalR2 have been established to have an antidepressant-like effect by enhancing the activities of selective serotonin reuptake inhibitors (SSRIs) and promoting the availability of transcription factors (64, 65). Importantly, recent research has reported that alarin, which is also localized in the brain areas linked with depression, like the medial amygdala and hypothalamus, has been demonstrated to have regulatory effects on depression-like behaviors in various animal models (11, 25–27, 66).

Wang and coworkers revealed for the first time that alarin exhibits potent antidepressant-like effects in both the acute stress model and the unpredictable chronic mild stress (UCMS) depression-like model. The study additionally demonstrated that alarin is distributed in the hypothalamus and LC, which are recognized as critical regions in mood-related disorders (26). Zhuang et al. consistently showed that central injection of alarin significantly ameliorates depression-like behaviors in the UCMS mouse model (28). Subsequently, the antidepressant properties of alarin were further confirmed by several other studies (11, 27, 66). However, the antidepressant-like effect of alarin has been shown to be dose-limited, with no remarkable effect at higher doses (at 2.0 nmol or higher). Although the mechanism by which alarin produces the antidepressant effect is not yet fully understood, a number of potential mechanisms have recently been reported. Here, some putative mechanisms of alarin-induced antidepressant actions are briefly discussed in two separate sections: the TrkB-mTOR signaling pathway and the hypothalamus–pituitary–adrenal (HPA) axis.

#### The tropomyosin-related kinase B-mammalian target of rapamycin signaling pathways

**FIGURE 1:**
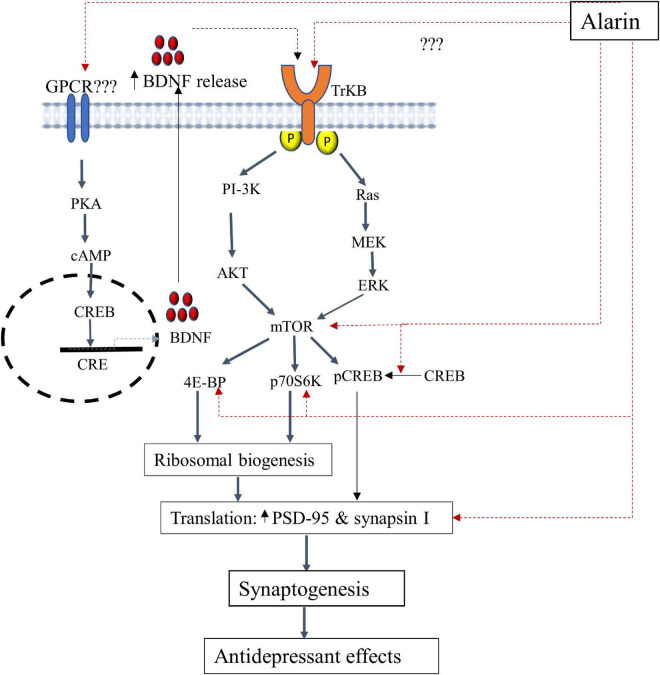
A schematic illustration of the putative antidepressant mechanism of alarin targeting the TrkB-mTOR signaling pathway and BDNF secretion. In this pathway, alarin may exert antidepressant-like effects by interacting with TrkB, possibly as a ligand, and stimulating downward signaling components like ERK and AKT. Besides, alarin may affect the activity of CREB by enhancing its phosphorylation. It also exhibits antidepressant effects by increasing mTOR, 4E-BP, p70S6K, PSD-95, and synapsin I. Moreover, alarin enhances the secretion of natural antidepressant peptide (BDNF) through a pathway that is not yet well defined. The synthesis and release of BDNF from the transcription process activates TrkB receptors and the downstream mTOR signaling pathway, which coincides with the therapeutic response. BDNF-or alarin-activated mTOR triggers the translational machinery, increasing synaptic protein (PSD-95 and synapsin I) synthesis involved in synaptogenesis to confer an antidepressant effect. BDNF, brain-derived neurotrophic factor; CREB, cAMP response element-binding protein; CRE, cAMP response element; ERK, extracellular signal-regulated kinase; mTOR, mammalian target of rapamycin; PI3K, phosphatidylinositol 3-kinase; p70S6K, ribosomal protein S6 kinase; PKA, protein kinase A; PSD-95, post-synaptic density 95; 4E-BP1, eukaryotic initiation factor 4E-binding protein 1; TrkB, tropomyosin-related kinase B.

According to several animal-based studies, alarin has been found to alleviate depression by targeting various components in the TrkB-mTOR signaling pathways as discussed below. The putative antidepressant mechanism of alarin using this pathway is further illustrated in Figure 1.

Tropomyosin-related kinase B receptor (TrkB): Alarin has been found to exert antidepressant-like effects by influencing tropomyosin-related kinase B (TrkB) which alters the downstream signaling pathways. TrkB is a member of the neurotrophic tyrosine kinase (NTRK) receptor family encoded by the *NTRK2* gene. TrkB has a high level of expression in the brain and serves as a common upstream receptor for a number of ligands such as the brain-derived neurotrophic factor (BDNF), neurotrophin 3 (NT3), and neurotrophin 4 (NT4) (67, 68). This receptor is crucial for neural cell differentiation, survival, and proliferation (69). Current research has shown that defective TrkB is closely linked with the pathophysiology of depression. This is evident from the significantly reduced TrkB expression in the prefrontal cortex and hippocampus of postmortem human subjects with major depression (70–72). Hence, alarin is postulated to have an effect similar to that of antidepressants by targeting TrkB receptor-mediated activation of downstream cascades, especially extracellular signal-regulated kinase (ERK) and protein kinase B (AKT) signaling pathways (11, 26, 27). TrkB receptor-mediated Ras-ERK and PI3K-AKT intracellular signaling pathways are involved in neuronal survival and neurogenesis and result in antidepressant-like effects (73). While the TrkB-activated Ras-ERK pathway is involved in cell proliferation, differentiation, apoptosis, and synaptic plasticity, the PI3K-AKT pathway is involved in the regulation of cell growth, survival, proliferation, and movement (74–77). Alarin is thus demonstrated to have antidepressant properties in UCMS-treated mice, likely by increasing the expression of AKT and ERK in some areas of the brain, including the prefrontal cortex, hippocampus, olfactory bulb, and hypothalamus. Despite the putative role of TrkB in mediating the antidepressant activities of alarin, additional research is needed to determine whether alarin directly acts on TrkB or whether TrkB itself is an unidentified receptor of alarin.

Mammalian target of rapamycin (mTOR) and downstream components: The mTOR is another target of alarin to achieve its antidepressant-like effects *via* ERK and AKT pathway activation. mTOR is a widely expressed serine/threonine kinase that belongs to the phosphatidylinositol 3-kinase (PI3K)-related kinase protein family and is a key player in controlling protein translation (78). It is a target of the ERK/AKT pathway that integrates signals from neuronal activity, growth factors, and nutrient levels to regulate the initiation of protein translation *via* its major downstream components, namely ribosomal protein S6 kinase (p70S6K) and eukaryotic initiation factor 4E-binding protein 1 (4E-BP1) (78, 79). Activation of mTOR phosphorylates p70S6K and 4E-BP and induces protein synthesis to regulate many integrated physiological functions of the nervous system, including neuronal development, synaptic plasticity, memory storage, and cognition (78, 80, 81). On the other hand, chronic stress disrupts mTOR signaling and markedly decreases synaptic proteins such as post-synaptic density 95 (PSD-95) and pre-synaptic protein synapsin I, indicating its role in the pathophysiology of depression (79, 82). The loss of synaptic proteins that play an important role in synaptic function (or plasticity) commonly results in impaired synaptic quantity and function, inducing neuronal changes, and increasing vulnerability to depression (83–85). The antidepressant-like effect of alarin may therefore be mediated by reactivating the mTOR signaling pathway, which will enhance synaptic protein synthesis and synaptic plasticity while reversing synaptic protein loss and impaired function (27). Increased synaptic expression of PSD-95 and synapsin I, by improving the development, maturation, and function of synapses, relieves depressive symptoms (82, 86–89). Intracerebroventricular (ICV) injection of alarin has also been proven to improve depression-like behaviors through the alarin-mediated restoration of UCMS-induced reduction of phospho-mTOR and phospho-4E-BP1 in the prefrontal cortex, hippocampus, hypothalamus, and olfactory bulb. Besides, alarin was observed to reverse UCMS-induced downregulations of p70S6K, PSD-95, and synapsin I expression in these brain regions (27). Additionally, alarin has been shown to affect the activity of the cAMP response element-binding protein (CREB), a downstream messenger of ERK and AKT. Acute alarin treatment has been reported to markedly increase phosphorylated CREB levels in the prefrontal cortex of stressed animals to basal levels of vehicle-treated mice, but no significant changes in CREB levels have been observed in other brain areas, such as the hippocampus, the olfactory bulb, and the hypothalamus (11). Several previous studies have indicated that rapid, long-lasting, potent antidepressant effects in both depressed patients and rodent models can be produced by increasing the activity of mTOR and the downstream signaling components that contribute to synaptic protein synthesis and synaptic plasticity (86, 90). This shows that alarin has pharmacological properties resembling those of antidepressant drugs and may be used as a treatment option for depression.

Brain-derived neurotrophic factor (BDNF): Alarin is also postulated to exhibit antidepressant effects through the upregulation of the expression of neurotrophin, particularly BDNF (26). BDNF is a dimeric protein in the neurotrophin family, which is thoroughly distributed in the brain, more abundantly in the cerebral cortex, hypothalamus, hippocampus, striatum, basal forebrain, and cerebellum (91). It promotes the growth, development, neuroprotection, and survival of neurons, as well as regulates neuronal apoptosis and increases synaptic plasticity. This ultimately prevents neuronal degeneration and death, which reveals the antidepressant effect of BDNF (67, 92). BDNF is suggested to exhibit a principal antidepressant role by fostering synaptic plasticity by acting as a ligand of the TrkB receptor, stimulating mTOR to induce synaptic protein synthesis (93–95). Therefore, alarin can potentially alleviate depression by up-expressing BDNF, as evidenced by the increased expression of BDNF mRNA in the prefrontal cortex and hippocampus of UCMS-treated mice after alarin administration (26). This action of alarin is similar to the effects of most antidepressants, which upregulate BDNF expression in rodent brain and human blood. The increase in endogenous BDNF, in turn, attenuates the symptoms of depression by reversing neuronal atrophy and promoting cell proliferation in the hippocampus and prefrontal cortex (96, 97). However, this alarin-induced BDNF upregulation is mediated by an unidentified receptor, even though it probably belongs to the GPCR receptor family.

#### The hypothalamic–pituitary–adrenal axis

**FIGURE 2:**
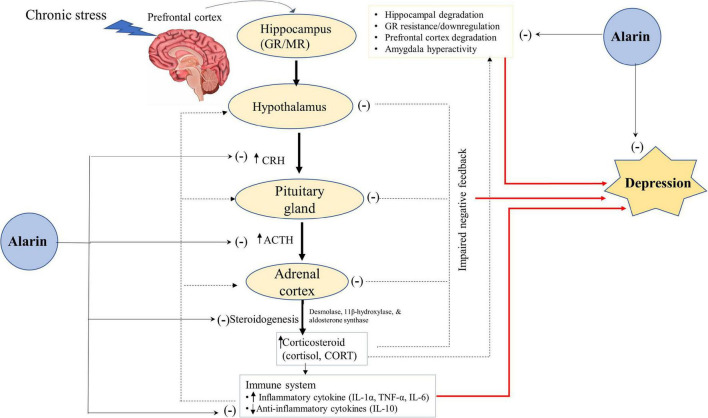
A hypothetical antidepressant activity of alarin through the regulation of the HPA-axis. Alarin relieves depression by acting as a hormone regulator that downregulates the activity of the HPA axis (CRH, ACTH, and Corticosteroids), and an enzyme inhibitor that decreases the expression of enzymes involved in steroidogenesis (desmolase, 11β-hydroxylase, and aldosterone synthase), thus lowering corticosteroid hormone synthesis. Besides, alarin alleviates depression by reducing HPA-axis overactivity resulting from increased pro-inflammatory cytokines (IL-1α, TNF-α, and IL-6) and decreased anti-inflammatory cytokines (IL-10). Alarin also has antidepressant properties due to its ability to restore hippocampal volume loss, GR resistance/downregulation, prefrontal cortex degradation, and amygdala hyperactivity. Arrow ends (→) indicate a stimulatory effect, while negative signs (–) show an inhibitory effect. ACTH, adrenocorticotropic hormone; CORT, corticosterone; CRH, corticotropin-releasing hormone; GR, glucocorticoid receptor; HPA, hypothalamic-pituitary-adrenal; IL, interleukin; MR, mineralocorticoid receptor; TNF, tumor necrosis factor.

Moreover, alarin regulates depression by controlling adrenocortical hormone secretion and the interaction of HPA axis organs (Figure 2). Like galanin, alarin plays a significant role in the regulation of depression-like behaviors *via* its antagonist activity against the hyperactivity of the HPA axis (93, 98). The abnormality of the HPA system, which is essential in stress responses and homeostasis maintenance, is involved in the pathophysiology of depression (57, 99, 100). It has been proposed that hyperactivity of the HPA axis and disruption of the stress feedback mechanism are common pathophysiological features of major depressive disorder in humans. A large amount of data suggests that aberrant stimulation of the HPA axis in depression activates the hormonal cascade in the brain (57). The hypothalamus secretes corticotropin-releasing hormone (CRH) in response to psychological stress in cortical brain regions. CRH stimulates the anterior pituitary gland to release corticotropin or adrenocorticotropic hormone (ACTH), which in turn triggers glucocorticoid secretion from the adrenal cortex to the plasma (91). In normal circumstances, the HPA axis is regulated to prevent it from becoming overactive by mineralocorticoid (MR) and glucocorticoid (GR) receptors. However, an imbalance in the MR/GR ratio, mainly a decrease in limbic GR receptor function and increased functional activity of the MR system, is seen in stress-related conditions like depression (101–103). Chronic stress in major depressive disorder induces neuronal degeneration in the hippocampus, leading to suppression and impairment of GRs that inhibit the negative feedback of glucocorticoids (104, 105). This disturbed stress feedback mechanism further activates the HPA axis and leads to the over-secretion of glucocorticoid levels (106–108).

Alarin may therefore have a crucial role in ameliorating depression by acting as a hormone regulator that downregulates the activity of the HPA axis. Available data shows that ICV injection of alarin in UCMS mice is associated with a decline in CRH expression levels in the hypothalamus, and a decrease in serum levels of CRH, ACTH, and corticosteroids [cortisol in humans and corticosterone (CORT) in animals] released from the HPA axis (26). Another study also found that alarin negatively affects the expression of enzymes involved in steroidogenesis, such as cholesterol desmolase, 11β-hydroxylase, and aldosterone synthase, in the adrenal glands. In addition, although it has no appreciable effect on blood concentrations of aldosterone and CORT, alarin substantially reduces blood hormone levels derived from the HPA axis, such as CRH, ACTH, and pro-opiomelanocortin (POMC) (66). This effect of alarin resembles those of other antidepressants that reduce the elevated levels of ACTH, cortisol, and CORT in depressed subjects (106–108). Collectively, alarin-mediated normalization of the serum levels of the key hormones of the HPA axis alleviates depression symptoms and exhibits an antidepressant-like effect.

Furthermore, the antidepressant-like properties of alarin may be mediated by restoring altered levels of the pro-inflammatory and anti-inflammatory cytokine, thereby reducing activation of the HPA axis (28). Based on numerous prior animal and human studies, inflammatory cytokines play an essential role in the pathophysiology of depression (109–112). Clinical data suggest that pro-inflammatory cytokines such as interleukin (IL)-1α, tumor necrosis factor (TNF)-α, and IL-6 disrupt GRs, activate the HPA axis, impair the central serotonin system, and consequently induce depressive symptoms (113). In contrast, anti-inflammatory cytokines are associated with reduced hyperactivity of the HPA axis and depressive symptomatology (28). Here, alarin reverses the UCMS-induced rise in pro-inflammatory cytokines (TNF-α and IL-6) and a decrease in the levels of anti-inflammatory cytokine (IL-10) in the blood, prefrontal cortex, hippocampus, and hypothalamus. In addition, alarin restores the UCMS-induced decrease in GR expression in these brain areas, which ultimately reduces HPA axis overactivity and depression (28).

### Potential therapeutic implication of alarin in depression

Based on existing theories on the etiology of depression, many antidepressant drugs have been discovered and utilized in the treatment of depression for a long time. A variety of antidepressant medications targeting these hypothetical pathomechanisms are currently under consideration for the treatment of depression (114). Nonetheless, inhibitors of monoamine transporters like SSRIs, NEP reuptake inhibitors (NRIs), and combined serotonin-NEP reuptake inhibitors (SNRIs) are nowadays noted as the most widely prescribed antidepressant medications (7). These medications attenuate a postulated deficiency of monoamine transmission by raising extrasynaptic NEP and/or serotonin levels (115). However, currently available antidepressant drugs raise concerns about their efficacy and adverse effects, increasing the necessity of developing appropriate antidepressants with new mechanisms. Hence, researchers are urged to work diligently on developing novel therapeutic drugs with efficient antidepressant effects and fewer side effects, as well as seeking better targets for antidepressant therapies to achieve and maintain remission.

Currently, numerous neuropeptides, including the galanin neuropeptide family and their receptors, are attracting researchers’ interest to use them as novel therapeutic targets for depressive disorders. This is because such neuropeptides colocalize with the neurotransmitters NEP, 5-HT, and DA in some brain areas and mediate anxiety and stress-related behavioral functions (8, 9). Hence, designing drugs that enhance peptidergic transmission, regulating the activity and functions of the co-expressing neurons, may serve as a promising treatment strategy for depression. Importantly, the parent peptide of the galanin family, galanin, has been reported to have remarkable therapeutic effects in depressed patients. The interaction of the serotonergic system and neuropeptides is a key aspect of galanin’s potential for treating major depression in rodents (10, 116, 117).

More interestingly, a large number of studies involving a variety of experimental rodent models have recently revealed that alarin exhibits potent antidepressant-like effects by employing several mechanisms. It has neuroprotective effects and prevent the occurrence of depression by upregulating the expression of TrkB, BDNF, and mTOR, as well as by downregulating the activity of the HPA axis (11, 26–28). As a consequence, it may become possible to derive therapeutic benefits from this new knowledge. Given its growing antidepressant role, alarin could be a peptide of the galanin family, in addition to galanin, that may be taken into consideration in the therapeutic arena of depression. Nowadays, it attracts researchers’ interest toward its potential implications in clinical practice as a treatment option for depression. But clinical studies regarding the pharmacological properties of alarin are still inadequate, warranting the need for further extensive clinical research. A better understanding of the antidepressant mechanisms of alarin may therefore make it possible to use it as an alternative treatment strategy for depression in clinical settings in the future. Overall, alarin has an intriguingly enormous potential for use in neuroscience research and novel drug development for depression, albeit its particular receptor remains to be determined.

## Arguments on alarin receptor

A large amount of data has demonstrated that the various physiological effects of the galanin family, such as galanin, the galanin message-associated peptide (GMAP), GALP, and spexin, are generally mediated by three subtypes of GalRs in the G protein-coupled receptors (GPCR) family, namely GAlR1, GalR2, and GalR3 (118). However, several receptor binding studies unveiled that alarin does not bind to any of the three GalRs (119). A study by Santic et al. reported that synthetic human alarin could not bind to membrane preparations of human neuroblastoma cells expressing GalR. It has low receptor binding affinity for GalRs, with a dissociation constant (Ki) >1,000 for GALR1, Ki >1,000 for GALR2, and Ki >10^6^ for GALR3 (13). Another study by Boughton et al. on hypothalamic explants further strengthened that alarin has no detectable affinity toward any of the three GalR subtypes (20). The inability of alarin to function *via* GalRs is proposed to be the result of the exclusion of exon three, leading to a loss of the GalR-binding domain necessary for the ability of other members of the galanin family to bind to GalRs (13). Thus, as alarin does not share any similarity with galanin and is not able to compete with radio-labeled galanin for known GalRs, alarin actions are unlikely to be mediated *via* such receptors (13, 15, 20).

Instead, the biological actions of alarin seem to be mediated *via* a distinct unknown receptor different from GalRs. In addition, evidence regarding the physiologic and pharmacologic characteristics of the alarin receptor is still insufficient. However, it has been indicated that alarin appears to mediate its effects through numerous, yet unidentified alarin-specific receptors other than GalRs (13, 19, 20). Fraley et al. demonstrated that unlike GALP, which is mediated by GalRs and is associated with motor deficits after ICV injection, central injection of alarin in mice is linked to the absence of motor impairments, supporting that alarin may act *via* a separate receptor (120, 121). It is also supported by another study that demonstrates that the alarin antagonist (the so-called Ala6-25Cys), but not the GalR blockers (M35 or C7), reverses the attenuating effects of alarin on myocardial infarction (MI)-induced cardiac fibrosis. Furthermore, this truncated alarin peptide (Ala6-25Cys) that missed the first five amino acids of the full-length alarin restores the inhibitory effects of alarin on angiotensin II-induced cardiac fibrosis (24, 122–124). Ala6-25Cys also antagonizes the acute orexigenic effects of centrally administered alarin (13, 19). This highlights that alarin produces biological effects that are unlikely to be mediated by the known GalRs, but rather by a specific receptor that has not yet been determined (24). But this unidentified alarin-specific receptor may or may not be other subtypes of GalRs that have not yet been known.

Although it is not yet known, many scholars have put forth their hypotheses about the unidentified alarin receptor and its possible category of receptor family. Santic et al. hypothesize that the unknown alarin receptors most likely belong to the GPCR receptor superfamily and that alarin may therefore function similarly to other members of the galanin family by employing GPCR signaling pathways (13). This speculation is based on the fact that the vascular system expresses a significant number of GPCRs and the majority of neuropeptide receptors are members of the GPCR receptor superfamily (13, 119). On the other hand, other studies argue that the receptors for alarin may belong to the broad family of receptor tyrosine kinases (RTKs). This hypothesis stems from the observation that many peptide receptors, including receptors for epidermal growth factor, vascular endothelial growth factor, and insulin, belong to the RTK family, which suggests that the receptor for alarin could belong to the RTKs. Additionally, the involvement of TrkB, an RTK, in alarin-mediated antidepressant-like effects, supports the notion that alarin-specific receptors may be a member of RTKs (11, 70–72).

Furthermore, there is some other information regarding the undiscovered receptor of alarin. Few studies showed unidentified alarin receptor-expressing nuclei in the murine brain, as evidenced by induction of Fos-ir expression after ICV injection of alarin in numerous hypothalamic regions that regulate food intake, body weight, and endocrine hormone secretion (16). Additionally, the existing data indicated that the N-terminal region of the alarin peptide would be crucial for binding with its undiscovered receptor, based on the ground that the first few amino acids of alarin are conserved between rodents and primates (26). Generally, the unknown receptors of alarin pose another challenge in identifying them based on the latest effects in pharmacology and biology as well as the mechanisms underlying these effects. Therefore, unraveling the type of receptor, the physiological activity, and the mechanism of action of alarin remain active areas of future research. The alarin antagonist (Ala6-25Cys), which lacks the first five amino acids of alarin and acts as a specific inhibitor of alarin actions, has been indicated to be a useful tool that could have paramount importance in the search for alarin-specific receptors (19).

## Conclusion and future directives

Depression is a mood disorder whose etiology has been explained by diverse proposed theories, but none of them has been able to adequately explain the nature of depression. Consequently, the effect of pharmacological modulation of some of the pathophysiological mechanisms of depression as an antidepressant therapy was disappointing. Besides, current widely used antidepressants that target monoamine transporters are reportedly associated with several limitations, like low therapeutic efficacy and side effects. Thus, discovering new targets and alternative antidepressants in depression treatment is required. Presently, multiple studies are ongoing to develop novel therapeutic strategies and replace the existing treatments for depression based on a more in-depth analysis of the behavioral and molecular mechanisms underpinning the disease.

Alarin, which is a 25-amino-acid long multifunctional neuropeptide, has recently gained recognition because its central administration is associated with the modulation of depressive-like behavior in animal models. Accumulated evidence shows that the expression of alarin in the brain, mainly in the hypothalamus and amygdala, is linked to its antidepressant-like effects. This reveals that alarin may potentially be used as a pharmacological agent for the treatment of depression, although extensive elucidation of its physiological or pharmacological characteristics is required. Alarin might also be of utmost significance in the antidepressant application by centrally altering alarin expression and/or activities using a medication that able to cross the blood brain barrier and modifies alarin’s effect. However, the details regarding antidepressant implications of alarin, including the effect of peripheral alarin administration and drugs that potentially modify the effect of alarin centrally, remain active area of research. Additionally, alarin may also be utilized in the future to treat depression that is resistant to standard antidepressant treatments, known as treatment-resistant depression. This could offer a solution to a major therapeutic challenge of treatment-resistant depression and greatly thrill mental health experts.

Nonetheless, data describing the alarin-specific receptors mediating the antidepressant effects and other biological activities are insufficient. A growing body of experimental evidence suggests that alarin is unlikely to bind to any known GalRs due to the absence of the GalR-binding domain. Besides, its particular receptor has not yet been determined, and this may open up a vast area of research to explore the unknown receptors for alarin. Overall, despite its promising therapeutic relevance, knowledge about the antidepressant effects of alarin is limited, so alarin is still in its infancy of use in the treatment of depression. This urges us to conduct more clinical research regarding the effects of alarin in depressed patients and its associated pharmacologic properties.

## Author contributions

All authors made a significant contribution to the work reported, whether that is in the conception, study design, execution, article search, or in all these areas; took part in drafting, revising or critically reviewing the article; gave final approval of the version to be published; have agreed on the journal to which the article has been submitted; and agreed to be accountable for all aspects of the work.

## Abbreviations

4E-BP1: 4E-binding protein 1
ACTH: adrenocorticotropic hormone
BDNF: brain-derived neurotrophic factor
CNS: central nervous system
CORT: corticosterone
CREB: cAMP response element-binding protein
CRH: corticotropin-releasing hormone
DA: dopamine
ERK: extracellular signal-regulated kinase
GALP: galanin-like peptide
GalR: galanin receptor
GMAP: galanin message-associated peptide
GPCR: G protein-coupled receptor
GR: glucocorticoid receptor
HPA: hypothalamus–pituitary–adrenal gland
ICV: intracerebroventricular
IL: interleukin
MAOIs: monoamine oxidase inhibitors
MI: myocardial infarction
MR: mineralocorticoid receptor
mTOR: mammalian target of rapamycin
NEP: norepinephrine
NMDA: N-methyl-D-aspartate
NRIs: NEP reuptake inhibitors
NTRK: neurotrophic tyrosine kinase
p70S6K: ribosomal protein S6 kinase
PI3K: phosphatidylinositol 3-kinase
POMC: pro-opiomelanocortin
PSD-95: post-synaptic density 95
RTKs: receptor tyrosine kinases
SNRIs: combined serotonin-NEP reuptake inhibitors
SSRIs: selective serotonin reuptake inhibitors
TCAs: tricyclic antidepressants
TNF: tumor necrosis factor
TrkB: tropomyosin-related kinase B
UCMS: unpredictable chronic mild stress.
